# Early Physical Rehabilitation after Sentinel Lymph Node Biopsy in Breast Cancer: Is It Feasible and Safe?

**DOI:** 10.3390/ijerph17228382

**Published:** 2020-11-12

**Authors:** Beatriz Ostos-Díaz, María Jesús Casuso-Holgado, María Jesús Muñoz-Fernández, Ana F. Carazo, Rocío Martín-Valero, Esther M. Medrano-Sánchez

**Affiliations:** 1 Department of Physiotherapy, University of Sevilla, 41009 Sevilla, Spain; beatrizostosdiaz@hotmail.es (B.O.-D.); mariamufe@hotmail.com (M.J.M.-F.); 2 Department of Physiotherapy, Faculty of Nursing, Physiotherapy and Podiatry, University of Seville, C/Avicena s/n, 41009 Seville, Spain; emedrano@us.es; 3 Department of Economy, Quantitative Methods and Economy History, Pablo de Olavide University, 41013 Sevilla, Spain; afercar@upo.es; 4 Department of Physiotherapy, Faculty of Health Sciences, University of Malaga, Arquitecto Francisco Peñalosa 3, Ampliación de Campus de Teatinos, 29071 Málaga, Spain; rovalemas@uma.es

**Keywords:** breast neoplasm, physical therapy specialty, sentinel lymph node biopsy, rehabilitation, health education

## Abstract

The primary purpose of this research was to investigate the feasibility and safety of delivering an early supervised physical therapy intervention to women after sentinel lymph node biopsy (SLNB); furthermore, we aimed to provide explorative data on its effects. This was a single-site feasibility study. Pre- and post-evaluation was conducted from baseline to follow-up at 6 months. Primary outcomes were participant recruitment, participant retention, compliance with the intervention, and safety. Secondary outcomes were shoulder range of motion, handgrip strength, upper limb pain and disability, scar recovery, quality of life, and the incidence of axillary web syndrome (AWS) and/or lymphoedema. A total of 43 participants (mean age 55.37 years) completed the trial and the follow-up period. A total of 91% of women who met the inclusion criteria agreed to participate, and the adherence rate was 80%. No adverse events were reported. Incidence of AWS was 9.3%, and there was no incidence of lymphoedema at 6 months. Our results support that this intervention is feasible and safe. The results presented in this study also provide preliminary evidence for the use of a rehabilitation program as a supportive intervention after SLNB, but future research on effectiveness is needed.

## 1. Introduction

Sentinel lymph node biopsy (SLNB) is endorsed as the recommended method for staging early breast cancer in clinically node-negative patients due to its benefits on arm morbidity compared with axillary lymph node dissection (ALND) [1,2]. Thus, when sentinel nodes are tumor-free, ALND is considered unnecessary [3,4]. Breast-conserving surgery is also the preferred local treatment option for the majority of early breast cancer patients [5], with the main goal of having a cosmetically acceptable outcome without compromising local tumor control [6].

Despite the overall trend towards SLNB in the early stages, this minimally invasive surgery can also lead to postoperative problems. The literature has confirmed a similar short-term impact on quality of life impact between ALND and SLNB surgery [7,8], and SLNB has been associated with morbidity of the upper limb even years after surgery [9,10]. Moreover, in a 1-year follow-up study, it was observed that 50% of node-negative patients had pain and impaired shoulder function, with 30% reporting an impaired range of shoulder motion [11].

Another of the most common conditions observed after breast cancer surgery is axillary web syndrome (AWS), described as a tight subcutaneous cord in the ipsilateral axilla [12]. A recent review summarized AWS incidence rates and concluded that although AWS is 1.7–7 times more frequent after ALND than after SLNB, the incidence reported in the literature for SLNB ranges from 0.9% to 41% [13]. Similarly, a recent systematic review demonstrated that the incidence of lymphoedema is still a problem in SLNB patients, mostly occurring 6–12 months after surgery [14].

The importance of early rehabilitation in the management of postsurgical complications in the upper limb after ALND and SLNB has been highlighted [15,16,17], but most research has focused on post-ALND physical therapy and/or educational programs [18,19,20,21,22]. Research investigating early rehabilitation after SLNB is scarce [23,24]. 

Scaffidi et al. [23] enrolled both ALND and SLNB patients in a program based on preoperative information, physiotherapy sessions during hospitalization, and self-care home rehabilitation. Results showed that at the 60-day follow-up examination there were no significant differences between the experimental and control groups. Moreover, 17.2% of women in the experimental group needed prescription of outpatient physical therapy at that moment. Similarly, Sato et al. [24] implemented an educational intervention before surgery for the prevention of side effects. Upper limb function and grip strength were significantly improved in the intervention group with ALND, but there was no significant improvement in the patients who underwent SLNB (Table S1 and Figure S1).

To date, no studies have reported on an early intervention supervised by physical therapists after hospital discharge in women undergoing SLNB. Taking into account this lack of previous research and the small benefits observed in this population when enrolled in self-care interventions, the aim of this study was to stablish the feasibility and safety of delivering an early supervised physical therapy and educational program in women after SLNB and to provide explorative data on its effects for a future randomized controlled trial.

## 2. Methods

### 2.1. Study Design and Setting

This was a single-site feasibility study. Data were collected for 15 months in the University Hospital Virgen del Rocio, Seville, Spain. Pre- and post-evaluation was conducted from baseline, with an intervention period of 2–4 weeks, and follow-up at 6 months. The research procedure was approved by the Andalusian Ethics Committee on Human Research (PEIBA Reference 1176-N-17).

### 2.2. Subjects

All participants involved in the study met the eligibility criteria. Inclusion criteria were women aged 18–90 years clinically diagnosed with breast cancer; undergoing SLNB surgery; without ipsilateral recurrence of breast cancer; medical authorization to participate; and verbal communication capabilities. Exclusion criteria were refusal to participate; failure to provide written informed consent; psychiatric disorders; a relevant systemic condition; previous upper limb surgery or an existing condition that limited shoulder movement; and any medical condition that may limit participation in the proposed treatment program.

All participants provided written informed consent and were provided with information on the study procedures.

### 2.3. Procedures

**Figure 1:**
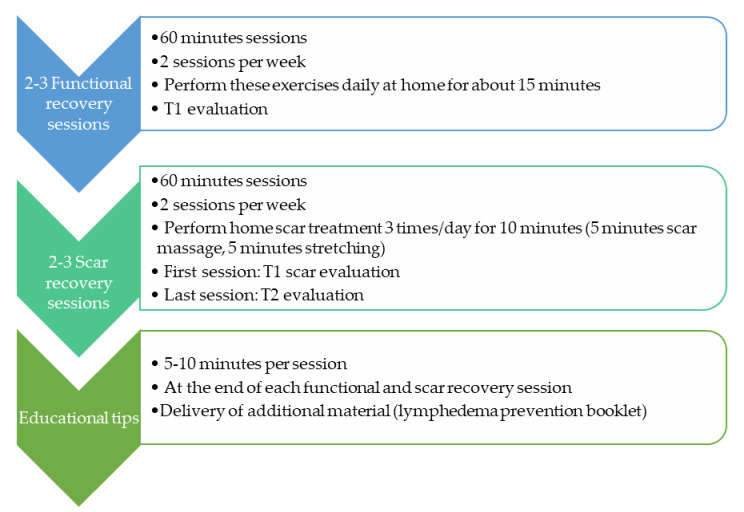
Temporal distribution of program sessions.

The researchers contacted patients scheduled for SLNB in the hospital, and all subjects who met the inclusion criteria were evaluated the day before surgery (T0 or baseline). Subjects were then enrolled in the intervention phase, which consisted of 4–6 sessions of physical therapy divided into three stages: functional recovery, scar recovery, and educational tips (Figure 1). At the beginning of the first functional recovery session, a second functional evaluation was performed; then, before the initiation of scar recovery, a scar and myofascial adhesion evaluation was performed (T1). In the last session, the final evaluation of all outcomes was performed (T2). Six months after surgery, the subjects were followed-up for lymphoedema evaluation (T3).

### 2.4. Intervention

An early physical therapy program in conjunction with educational tips was carried out. One month after the initial session, the patient should have completed the program, which included 4–6 sessions divided into three phases as follows. This temporal distribution was agreed with the surgeons, aiming to conclude our intervention prior to the first post-surgery revision. Two sessions per week were initially planned for all subjects, but in case of fear of movement, important shoulder restrictions or scar adhesions, extra sessions were delivered until a maximum of six sessions. 

#### 2.4.1. Functional Recovery

The objective of this stage was to use exercises to maintain the flexibility and elasticity of the muscles surrounding the shoulder joint, to facilitate lymphatic flow, and to maintain complete shoulder range of motion (ROM). These exercises were also intended to decrease pain and to prepare patients for daily activities.

The program of exercises was based on respiratory movements, particularly diaphragmatic breathing, accompanied by soft upper limb movements. Four exercises were undertaken in a sitting position: (i) hands in the abdominal region while inhaling and then performing a movement of 90° shoulder abduction and elbow extension in the frontal plane during exhalation; (ii) interlocking the hands and performing a shoulder flexion movement with extension of the elbows to chest level while inhaling; (iii) the same as the second exercise but achieving maximum possible shoulder flexion in the sagittal plane; (iv) 90° shoulder flexion with the forearm on the stretcher and trying to touch the knees with the abdomen while breathing out.

Moreover, patients were instructed to perform these exercises daily for about 15 min.

#### 2.4.2. Scar Recovery

The first session of scar recovery took place at least 2 days after the removal of stitches. The objective of this phase was to normalize the scar area while avoiding complications such as hypertrophic or retractile scarring, pain, or tissue clamps.

First, patients were instructed how to clean the scar in order to gently eliminate scabbing and reduce marks (i.e., showering, drying, and applying an antiseptic and Vaseline). Then, they were shown how to normalize the surrounding scar area by using massage therapy and kinesiotherapy, with the aim of giving elasticity and avoiding adhesions. Emphasis was put on hardened areas. To initiate scar normalization, patients mobilized their affected breast in all directions with the non-affected hand. Furthermore, the physical therapist gave instructions on how to self-massage the scar: two fingers located above the scar, moving down and then up in order to raise the subcutaneous tissue. 

**Figure 2:**
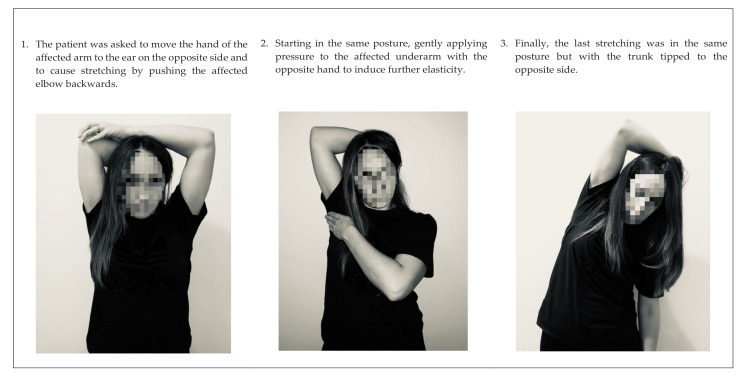
Stretching exercises in the scar recovery phase.

To end these sessions, underarm stretching was performed, keeping in mind that there may be discomfort but not pain. The patient was asked to move the hand of the affected arm to the ear on the opposite side and to cause stretching by pushing the affected elbow backwards or, starting in the same posture, gently applying pressure to the affected underarm with the opposite hand to induce further elasticity. Finally, the last stretching was in the same posture but with the trunk tipped to the opposite side. Patients were asked to maintain each position for 45–60 seconds, repeating it three times (Figure 2). They were also asked to perform scar treatment at home three times a day for 10 min (5 min scar massage, 5 min stretching).

Both the functional and scar sessions lasted approximately 60 min. In these sessions, the physical therapists ensured that all the patients were competent with the treatment procedures.

#### 2.4.3. Educational Tips

Throughout the functional and scar sessions, educational tips centered on lymphoedema prevention (i.e., an adequate bra that fits but does not press excessively) and postural hygiene were given to the participants. They were provided with information on how to improve the lymphatic system, as well as how to avoid risks that can contribute to its depletion. Both verbal and graphic information was used. Table S1 and Figure S1 are available online.

### 2.5. Outcomes

#### 2.5.1. Primary Outcomes

Feasibility of the procedure and intervention proposed were assessed by participant recruitment, participant retention, and compliance with the intervention. Safety was assessed via adverse events registry. 

#### 2.5.2. Secondary Outcomes

Although feasibility studies cannot answer questions about the effectiveness or efficacy of an intervention, preliminary data about clinical outcomes could be of interest for the design of a future clinical controlled trial. The following physical outcomes were assessed.

##### Shoulder Range of Motion and Handgrip Strength

Active range of motion (ROM) of the shoulder (flexion, extension, abduction, and internal and external rotation) was assessed in a sitting position. If an active ROM deficit was identified, the subject was supine and the ROM deficit was recorded with a conventional goniometer. A single index was calculated as the percentage of the global movement [25].

Handgrip strength was assessed with a Jamar digital dynamometer, which provides accurate measurements up to 90 kg and has a calibration system to secure reliability. Participants were seated with their elbow flexed at a 90° angle, and they performed three maximal contractions with the ipsilateral hand. The average score of these three measurements was then recorded [26].

##### Clinical Signs

Upper limb pain and disability were measured with the Shoulder Pain and Disability Index (SPADI) [27,28]. Subjects were asked to report their pain and disability on the subscales for different situations, ranging from 0 (no pain/disability) to 10 (worst pain ever experienced/disability). Total scores range from 0 to 130. A higher score indicates greater pain-related disability. The minimum detectable change (MDC) for the Spanish version has been established at 12.2% [27].

##### Quality of Life

Quality of life was evaluated with the EORT QLQ-BR23 questionnaire [29], composed of 23 items divided into four functional scales (body image, sexual functioning, sexual enjoyment, future perspective) and four symptom scales (systematic therapy side effects, breast symptoms, arm symptoms, and upset by hair loss). Scores vary from 0 (worst) to 100 (best) for function and from 0 (best) to 100 (worst) for symptoms. A higher score indicates better functioning and more symptoms. A 5-point difference in quality of life scores is considered the minimum clinically significant difference [30].

##### Scar

The state of the scar was assessed with the Patient and Observer Scar Assessment Scale (POSAS) [31]. This consists of six parameters for the evaluation of scars: vascularity, pigmentation, thickness, relief, pliability, and surface area. Each parameter uses a 0–10 points scoring system, with 0 representing normal skin and 10 representing the worst scar. Total scores range from 0 to 60; the higher the score, the worse the condition of the scar.

##### Myofascial Adhesions

The evaluation of myofascial adhesions was measured with the Myofascial Adhesions in Patients after Breast Cancer (MAP-BC) tool [32], which evaluates the degree of myofascial adhesions at seven anatomical locations in breast cancer patients. At each location, the degree of myofascial adhesions is scored at three levels of depth (skin, superficial, and deep) on a 4-point scale (between no adhesions and very strong adhesions), with 0 for no restriction in tissue gliding and 3 for when tissue gliding is virtually impossible. Total scores range from 0 to 63. A higher score indicates greater myofascial adhesions.

##### Axillary Web Syndrome and Lymphoedema

The presence of AWS was evaluated by observation and palpation. The physical examination was performed in the manner suggested in previous research: the patient in a supine position with the elbow extended and the shoulder maximally abducted. The evaluator visualizes and palpates for cords (including the axilla) down the upper arm from the axilla to, and across, the antecubital space and down the forearm to the base of the thumb [12,33].

The presence of lymphoedema was evaluated by telephone survey 6 months after surgery. Women were classified as having self-reported lymphoedema if they answered ‘yes’ to the question “Since your breast cancer surgery, has a doctor ever told you that you have lymphoedema or arm oedema?” [34].

This evaluation and the intervention detailed below were performed by two physical therapists with at least 3 years of experience in the breast cancer rehabilitation unit. Before initiating the study, consensus meetings were held, and the same materials were used to ensure that both physical therapists implemented the same interventions.

### 2.6. Data Analysis and Statistics

Data were grouped according to patient categories and screened for any obvious errors, anomalies, and duplications within each set. These data were then subjected to the Shapiro-Wilk test to determine whether there was a normal distribution. As there was no normal distribution, a non-parametric test was used. The Wilcoxon test was used to analyze within-group differences before and after treatment. For better results comprehension, measurements of central tendency and dispersion were also calculated for all outcome measurements, in addition to median, minimum, and maximum scores. Effect size was calculated using the statistic r (Rosenthal). We adopted *p* < 0.05 as the statistical significance limit. Data analysis was carried out using the Statistical Package for the Social Sciences (SPSS), version 17.0 ((IBM Corp, Armonk, NY, USA), and GraphPad Prism version 8 (GraphPad Inc, San Diego, CA, USA) was used to construct the plots. 

## 3. Results

### 3.1. Recruitment

**Figure 3:**
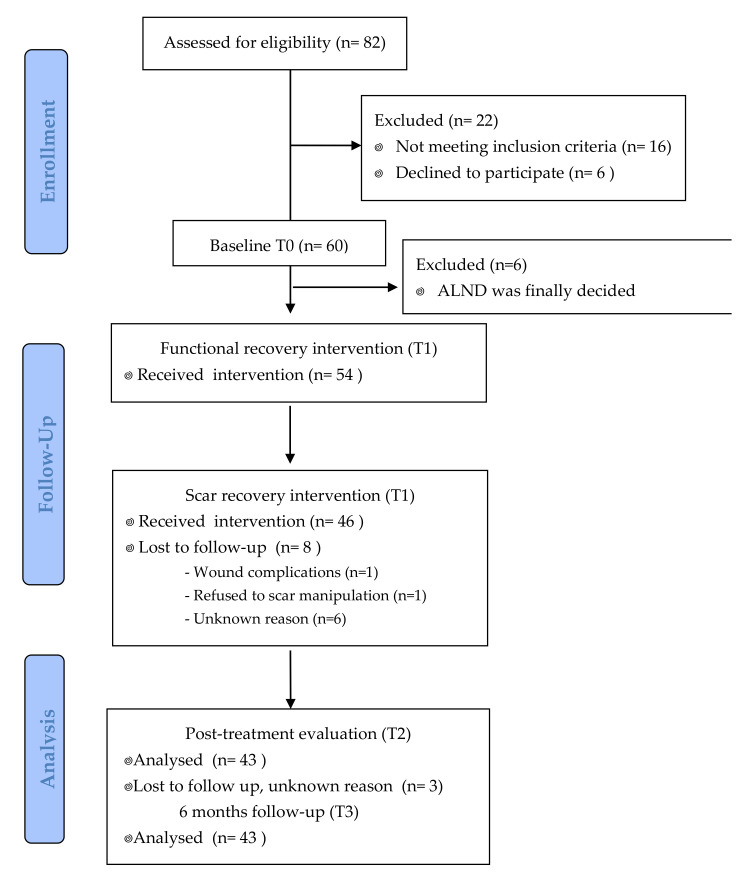
Patient flow chart.

During the study period, 82 patients were scheduled for SLNB surgery. Of these, 16 subjects were excluded based on exclusion criteria and 6 patients declined to participate in the study. The main reason for declining was the amount of time involved in travelling to the hospital. After surgery, six more participants had to be excluded due to ALND being finally carried out. We observed a recruitment rate of four participants per month, and 91% of women who met the inclusion criteria accepted to participate (Figure 3).

### 3.2. Adherence and Compliance

Initially, 60 women were assessed at baseline, and 54 of them met the inclusion criteria. All the treatment sessions were attended by 46 patients, of whom 3 were lost to evaluation at T2 and to follow-up at 6 months (unknown reason). Thus, the rate of adherence to the intervention was 80%, as can be observed in Figure 3. At the beginning of scar recovery sessions, 2 women refused to continue due to wound complications or fear of scar manipulation, and 9 women dropped out for unknown reasons. The compliance with the intervention was excellent, and all participants who completed the trial attended all sessions. 

The mean time to begin the early physical therapy program was 9.38 days. The mean duration of the intervention was 23.16 days, distributed over a mean of 3.41 weeks and with a mean number of sessions of 4.09 (Table 1).

### 3.3. Safety

Some participants reported discomfort along the upper limb and armpit when performing the functional recovery exercises. This was identified as tissue tension but not pain. One participant reported wound infection, but it happened before starting scar treatment. No serious adverse events were observed.

### 3.4. Preliminary Data of Clinical Outcomes

The mean age of the sample was 55.37 years. Approximately half of the subjects had an overweight body mass index (BMI) of 25–29.9 kg/m² (24% were classified as obese at ≥30 kg/m²). The majority of subjects (86%) underwent breast conserving surgery, 14% had a simple mastectomy, and 30% received adjuvant therapy along with surgery (radiotherapy, 5%; chemotherapy, 23%). A detailed description of the sample and intervention characteristics is provided in Table 1.

**Figure 4:**
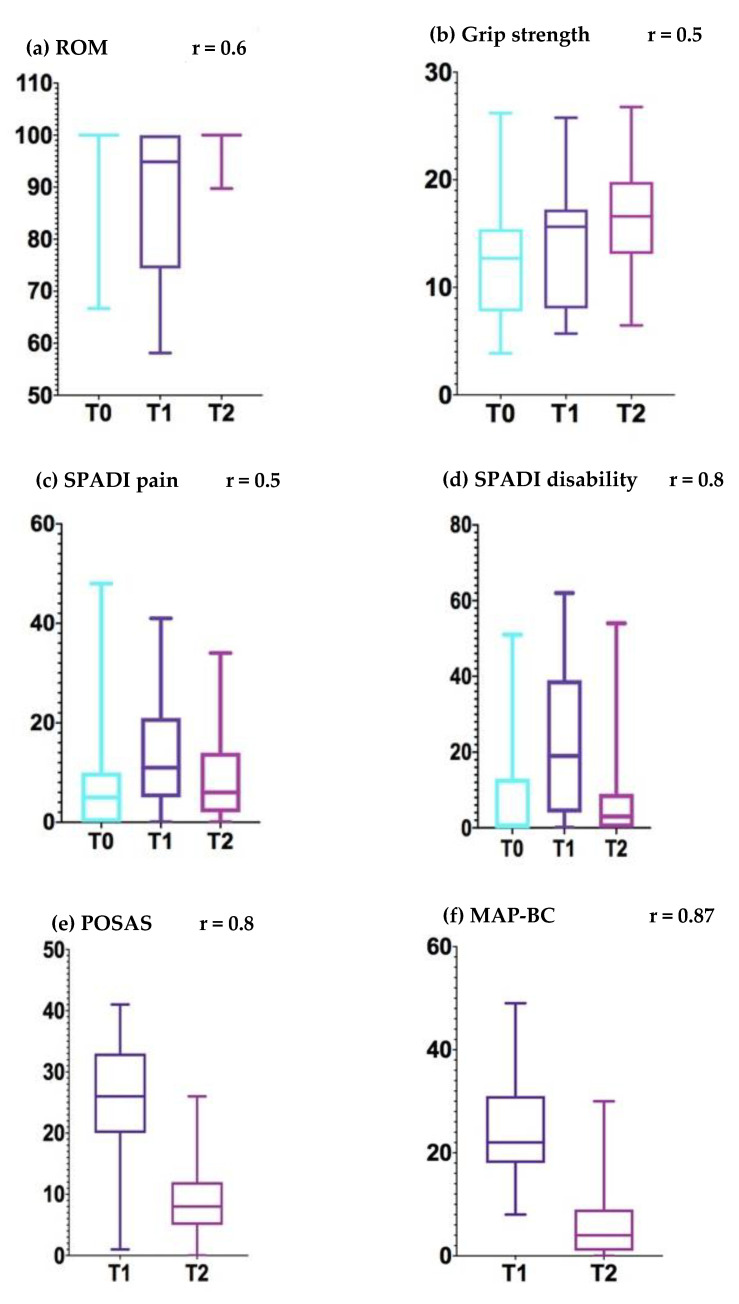
Outcome graphical representation in boxplots: (**a**) range of motion, (**b**) handgrip strength, (**c**) shoulder pain, (**d**) shoulder disability, (**e**) state of scar, and (**f**) myofascial adhesions. Effect size (**r**).

In comparison with baseline (T0), significant differences were found in the postsurgical evaluation of shoulder ROM (*p* < 0.001), handgrip strength (*p* < 0.05), shoulder pain (*p* < 0.05), shoulder disability (*p* < 0.001), and global symptoms (*p* < 0.001). After the intervention (T2), significant differences (*p* < 0.001) were found in shoulder ROM (r = 0.6), handgrip strength (r = 0.5), shoulder pain (r = 0.5), shoulder disability (r = 0.8), state of scar (r = 0.8), and myofascial adhesions (r = 0.87) in comparison with the postsurgical evaluation (T1) (Figure 4). Effects sizes were moderate to high for these variables. Furthermore, at the end of the intervention (T2), shoulder pain, shoulder disability, global function, and global symptoms were close to baseline scores. In addition, shoulder ROM and handgrip strength improved (*p* < 0.05). Incidence of AWS was 9.3%, and the incidence of lymphoedema at 6 months was 0% (Table 2).

## 4. Discussion

This is the first study to report on the feasibility and safety of an early supervised physical therapy program plus educational tips in women after sentinel lymph node biopsy. Our results suggest that this intervention is feasible and safe. Over 15 months, a total of 60 women were recruited, and 43 completed the trial. The mean duration of the rehabilitation program was 3 weeks, and participants received a mean of four sessions. No serious adverse events were reported, and the compliance with the intervention was excellent. This information could help to determine anticipated recruitment and adherence for a larger trial.

The results presented in this study also provide preliminary evidence for the use of a rehabilitation program as a supportive intervention after SLNB. As it was expected, in the short term, SLNB surgery had a negative physical impact in women. After the intervention, shoulder pain, shoulder disability, global function, and global symptoms were restored to baseline. Furthermore, shoulder ROM and handgrip strength were significantly high. 

Our results are in disagreement with previous research about the natural course of the recovery after SLNB. Kootstra et al. [35] reported functional limitations 6 weeks after surgery with decreased shoulder range of motion, shoulder strength, and handgrip strength. Similarly, Rönkä et al. [36] observed shoulder motion restriction in flexion and abduction 2 weeks after surgery (37% and 44% of the participants, respectively). De Groef et al. [11] showed that 1 year after surgery, 50% of sentinel node-negative breast cancer patients had pain, about 30% had decreased ROM, 8% had decreased handgrip strength, and 49% presented disability. Liu et al. [37] also reported that 1 year after surgery, the prevalence of pain in sentinel node-negative patients ranged from 8.0% to 36.0%, the prevalence of restricted range of motion of the affected arm varied from 6.4% to 31%, and the prevalence of grip strength reduction ranged from 17.0% to 19.0%. They concluded that SLNB-associated morbidity was a clinical problem. Similarly, Langer et al. [38] reported that the morbidity after SLNB was not negligible. They observed that in an intermediate-term follow up, 3.7% of women had painful scars and 1.6% keloid scars. 

These results indicate that breast surgery using the SLNB technique may also cause short-term and long-term adverse effects; nevertheless, in our study, these variables were restored to baseline at 1 month after surgery.

At 6 months we observed no lymphoedema incidence in node-negative cases. The overall rate of lymphoedema incidence reported in the literature for this population is quite broad, ranging from 0% to 63.4% [14]. For this reason, it is only possible to suggest that the intervention could help to prevent lymphoedema after SLNB. Another important side effect associated with breast cancer surgery is AWS, which most frequently becomes symptomatic between 2 and 8 weeks postoperatively [12]. Across different studies, the incidence of AWS after SLNB varies widely from 0.9% to 41% [13,39]. We observed an incidence rate of 9.3%, but due to the wide range reported in the literature, it is inconclusive whether the intervention proposed might prevent this syndrome. Taking into account these preliminary data, our results seem to be in accordance with previous research supporting the use of physical therapy to reduce the severity of ROM, symptoms, and functional upper extremity limitations [40], although further research is needed.

Concerning the intervention, the present study implemented a protocol with three components, namely functional recovery, scar recovery, and educational tips, distributed over a minimum of four and a maximum of six sessions supervised by a physical therapist. Previous research has only reported the implementation of educational programs based on preoperative information regarding possible side effects and education on the precautions after surgery [23,24] and physical therapy sessions during hospitalization [23]. When patients were discharged, they were encouraged to perform self-care. This is the first research implementing a supervised physical therapy intervention after hospital discharge and also the first protocol that includes scar treatment. 

### 4.1. Study Limitations

First, a methodological restriction is implicit in single-group pre- and post-test studies. However, this feasibility study approaches a novel intervention, and its preliminary results support a future randomized controlled trial. The pre-surgery evaluation at baseline could be assumed as women´s own controls. Second, lymphoedema surveillance at 6 months of follow-up was only self-reported. Finally, because upper limb impairments have been reported in node-negative patients after 1 year, a longer follow-up period for these outcomes would be desirable. 

### 4.2. Study Strengths

This is the first study to investigate the feasibility and safety of an early supervised physical therapy program after SLNB. This is also the first study that develops a program with three components (functional recovery, scar recovery, and educational tips) for this population. Second, our preliminary results suggest that a brief physical therapy and educational program might prevent morbidity associated with surgery, but future research is needed.

## 5. Conclusions

An early supervised physical therapy program plus educational tips after SLNB is feasible and safe. High recruitment and adherence rates were reported (91% and 80%, respectively), and no serious adverse events were observed. Preliminary evidence based on effect sizes (r = 0.5–0.87) suggest that it would be reasonable to integrate this intervention in a post-surgery model of care, but future controlled clinical trial is needed. With regard to the results and the proposed brief intervention, a cost-effectiveness study would be also of interest.

## Figures and Tables

**Table 1:** Subjects´ demographics and clinical-surgical characteristics.

Demographics and Characteristics	Number of Subjects = 43
**Age at Diagnosis, years**	Mean ± SD: 55.37 ±10.7 Minimum: 31 Maximum: 71
<45	7 (16.28%)
45–54	13 (30.23%)
55–64	11 (25.58%)
+65	12 (27.90%)
**Body Mass Index, Baseline, kg/m^2^ (N = 42)**	Mean ± SD: 27.14 ± 6.08 Minimum: 17.97 Maximum: 49.33
Normal: 18.5−24.9	15 (35.71%)
Overweight: 25–29.9	17 (40.47%)
Obese: ≥ 30	10 (23.80%)
**Ethnicity**	
Caucasian	40 (93%)
African	1 (2.3%)
Latin American	2 (4.7%)
**Type of Breast Cancer**	
Ductal carcinoma in situ (DCIS)	6 (14%)
Invasive Ductal Carcinoma (IDC)	36 (83.7%)
Lobular carcinoma in situ	1 (2.3%)
**Stage of Breast Cancer**	
0	6 (15.8%)
IA	23 (60.5%)
IIA	4 (10.5%)
IB	2 (5.3%)
IIB	3 (7.9%)
**Type of breast surgery**	
Simple unilateral mastectomy	6 (14%)
Breast conserving surgery	37 (86%)
**Numbers of lymph nodes removed**	Mean ± SD: 2.19 ± 1.18 Minimum: 1 Maximum:6
**Positive Lymph Nodes**	
Yes	6 (14%)
No	37 (86%)
**Side Involved**	
Right	23 (53.5%)
Left	19 (44.2%)
Bilateral	1 (2.3%)
**Involved side to hand dominance**	
Ipsilateral side	24 (55.8%)
Contralateral side	19 (44.2%)
**Adjuvant Therapy**	
Yes	13 (30.2%)
No	30 (69.8%)
**Type of Adjuvant Therapy**	
Radiation	2 (4.7%)
Chemotherapy	10 (23.3%)
**Numbers of sessions of adjuvant therapy**	Numbers of subjects = 13 (30.23) Mean ± SD: 11.46 ± 4.29 Minimum: 6 Maximum:18
**Days to early physical therapy program**	Mean ±SD: 9.38 ± 4.50 Minimum: 5 Maximum: 26
**Duration (days) of physical therapy program**	Mean ± SD: 23.16 ± 5.15 Minimum: 14 Maximum: 30
**Numbers of sessions of physical therapy**	Mean ± SD: 4.09 ±0.366 Minimum: 4 Maximum: 6

**Table 2:** Outcome measures for participants at baseline, post-surgery, and post-intervention.

Outcomes	T0 Mean ± SD Med [min, max]	T1 Mean ± SD Med [min, max]	T2 Mean ± SD Med [min, max]		T1-T0 *p*-Value	T2-T1 *p*-Value	T2-T0 *p*-Value
Global shoulder ROM (% index)	96.81 ± 8.13 100 [66.67, 100]	87.95 ± 13.54 94.87 [58.12, 100]	99.76 ± 1.56 100 [89.74, 100]		**0 < 001** T1 < T0	**0 < 001** T2 > T1	**0.012** T2 > T0
Grip strength (Kg)	12.48 ± 5.11 12.70 [3.86, 26.20]	14.36 ± 5 15.63 [5.7, 25.26]	16.56 ± 4.71 16.60 [6.46, 26.76]		**0.005** T1 > T0	**0.001** T2 > T1	**0 < 001** T2 > T0
Shoulder pain (SPADI 0–50 points)	8.27 ± 11.86 5 [0, 48]	13.23 ± 10.27 11 [0,41]	8.58 ± 8.23 6 [0,34]		**0.014** T1 > T0	**0.001** T2 < T1	0.678 T2 > T0
Shoulder disability (SPADI 0–80 points)	7.09 ± 12.43 0 [0, 51]	21.97 ± 19.41 19 [0, 62]	7.69 ± 12.45 3 [0, 54]		**0 < 001** T1 > T0	**0 < 001** T2 < T1	0.519 T2 > T0
Global EORTC QLQ-BR23 function ↑ (0–100 points)	54.89 ± 16.75 55.55 [0, 91.67]	52.29 ± 15.35 55.55 [0, 77.08]	52.92 ± 16.22 55.55 [0, 83.33]		0.380 T1 < T0	0.761 T2 < T1	0.601 T2 < T0
Global EORTC QLQ-BR23 symptoms ↓ (0–100 points)	14.32 ± 16.55 8.73 [0, 70.54]	20.40 ± 12.83 19.44 [2.78, 60.81]	18.64 ± 11.80 15.74 [2.78, 50.53]		**0 < 001** T1 > T0	0.223 T2 < T1	0.14 T2 > T0
State of scar (POSAS 0–60 points)		24.74 ± 10.15 26 [1, 41]	9.53 ± 6.19 8 [0, 26]		-	**0 < 001** T2 < T1	-
Myofascial adhesions (MAP-BC 0–63 points)		24.74 ± 10.37 22 [8, 49]	5.62 ± 5.86 4 [0, 30]		-	**0 < 001** T2 < T1	-
Axillary web syndrome (%)			Yes	4(9.3)	-	-	-
			No	39(90.7)	-	-	-
Lymphedema (%)			Yes	None	-	-	-
			No	43 (100)	-	-	-

SD: standard deviation; med [min-max]: median [minimum-maximum]; T0: baseline, T1: post-surgery, T2: post-intervention; ↑ EORTC QLQ-BR23 functioning scales, higher scores indicate better functioning.; ↓ EORTC QLQ-BR23 symptoms scales, higher scores indicate more problems. MAP-BC: Myofascial Adhesions in Patients after Breast Cancer tool, POSAS: Patient and Observer Scar Assessment Scale, SPADI: Shoulder Pain and Disability Index. Bold text indicates significant differences.

## Supplementary Materials

The following are available online at https://www.mdpi.com/1660-4601/17/22/8382/s1, Table S1: Comparison with previous research about post-surgery rehabilitation in breast cancer, Figure S1: educational tips booklet.
